# Vertical Suspension Optimization for a High-Speed Train with PSO Intelligent Method

**DOI:** 10.1155/2021/1526792

**Published:** 2021-10-21

**Authors:** Zhongcheng Qiu, Shichang Han, Jing Na, Chen Wang

**Affiliations:** ^1^Faculty of Mechanical and Electrical Engineering, Kunming University of Science and Technology, 650500 Kunming, China; ^2^Yunnan Dahongshan Pipeline Co., Ltd., 650302 Kunming, China

## Abstract

Intelligent methods and algorithms have promoted the development of the intelligent transportation system in many ways. In the rail transportation, the vertical performance of a high-speed train suspension system has a great impact on the riding comfort of the train. Based on the intelligent optimization method of particle swarm optimization (PSO) algorithm, different inerter-spring-damper (ISD) suspension layouts are proposed for better riding comfort. A 10-degree-of-freedom (10-DOF) vertical dynamic model of a high-speed train is established, and the new suspension layouts are applied to the primary and secondary suspension of the train at the same time. Optimizations are carried out for the suspension parameters of the high-speed train. Performances of different suspension layouts at different running speeds are analysed and compared. The best layout for suspension is concluded. What is more, the virtual prototype simulation and analysis of a high-speed train with consideration of nonlinear inerters are carried out. Friction of a rack-pinion inerter is simulated in the virtual prototype simulation. And the influence of nonlinearity is discussed compared with the ideal suspensions. All the results can represent a guidance for future train suspension design and help the intelligent rail transportation system to be more comfortable.

## 1. Introduction

With the development of the modern technology, intelligent transportation system has attracted more and more attentions all over the world. It is the product of the integration of intelligent technology and modern transportation system [1]. With the support of the intelligent technology and algorithm, the transportation management, operating pattern, service mode, and the vehicle design of transportation have been safer, more efficient, and environmental friendly.

For the intelligent transportation management, with wireless sensor networks technology, Chakraborty et al. [2] propose an algorithm to determine the green light duration for dispatching of emergency vehicle. Li et al. [3] put forward the optimization idea of Beijing road traffic law enforcement business process by using information technology, which provides reference to develop the traffic law enforcement business of the Beijing intelligent traffic management system. The operating pattern and service mode of transportation also developed with intelligent technology. In study [4], the Bayesian network theory is used to establish a traffic light independent intelligent decision model, which realize online reasoning and determine the best traffic light time according to the real-time dynamic information of traffic conditions. Based on the cellular automation model in micro perspective, Chen et al. [5] study the influence of an intelligent traffic system with mixed autonomous vehicles and human-driven vehicles with foresight degree of autonomous vehicles, the ratio of autonomous vehicles to human-driven vehicles, vehicle density, and the probability of random deceleration of human-driven vehicles. In order to mitigate and avoid traffic congestion, traffic prediction and optimal routing are researched by many intelligent methods, such as machine-learning-based method [6], ant colony algorithm [7], and neural network [8, 9]. It can be seen that intelligent methods play a more and more important role in developing intelligent transportation system.

The promotion of vehicle design with intelligent technology is also of significance. In the rail traffic, for the controller design, Sun et al. [10] have taken maglev train as the research object and designed a neural network-based supervisor controller (NNBSC), which realize effective control for the scenarios of random disturbance force, flexible track, and time-delay of maglev vehicle systems. Then a semi-supervised controller based on deep belief network (DBN) algorithm and the output-constrained controller are presented, research indicates that the controller can ensure the vertical security of maglev train [11]. On the other hand, for the vibration attenuation control and suspension design, many researchers report their achievements in improving suspensions of the train to enhance the performance. Intelligent algorithms such as genetic algorithm (GA) [12, 13] and particle swarm optimization (PSO) [14] are widely used in the research of suspension system. In particular, with the proposal of inerter by Professor Smith of Cambridge University [15], a new type of suspension named inerter-spring-damper (ISD) suspension is presented. And intelligent algorithms still play an important role in parameters optimization of ISD suspensions. Li et al. [12] use genetic algorithm to optimize the suspension parameters. Yu et al. [13] use multi-island genetic algorithm to optimize the parameters of the vertical suspension system of the high-speed train, and the riding comfort is improved significantly after optimization. Sun et al. [14] propose four suspension layouts with inerter and use particle swarm optimization algorithm to optimize the parameters, which is verified by simulation on a 6-DOF rail vehicle model. The results show that the parameters obtained by the optimization algorithm can effectively improve the performance of suspension system. All the researches have proven that inerters can be an efficient passive way to serve the suspension improvement in the intelligent rail traffic system.

However, few studies on the suspensions of the high-speed train with inerters are reported, which is still worthy to be explored. What is more, because of the high speed, the safety and cost of the experiment, most studies are focused on the theoretical analysis. This paper firstly introduces four different layouts with inerters to the suspensions of a high-speed train; vertical performances are studied at the high speed of 300 km/h, 350 km/h, and 380 km/h. Then, virtual prototype technology is introduced to carry out the running performance simulation of the high-speed train, which contains friction of the inerters as nonlinearity. The main contributions of this work are summarized as follows:

1. One ISD layout is proposed for the suspension of a high-speed train, which benefits the vertical performance of the riding comfort at different speeds.
2. Virtual prototype of the high-speed train equipped with the proposed ISD suspension is established. Friction of the inerters as nonlinearity is studied to examine the influence of the performance.

This paper is arranged as follows: in Section 2, a 10-DOF vertical high-speed train is modelled. And the systems combined with five suspension layouts are described. In Section 3, the intelligent algorithm of PSO is introduced and optimization of suspension parameters is explored.  Section 4 shows the performance of different train suspensions at different running speeds. Comparisons are discussed in both time and frequency domain. And the best layout of the suspension is concluded. In Section 5, a virtual prototype of the high-speed train is employed to research the influence of the inerters with frictions in consideration. Performance is compared with the ideal suspension. Finally, some conclusions are drawn in Section 6.

## 2. Modelling of the High-Speed Train System

In this section, a vertical 10-DOF high-speed train model is modelled and five basic suspension layouts are introduced. Dynamic equations of the system with different layouts are established as well.

### 2.1. Vertical Dynamic Model

**Figure 1:**
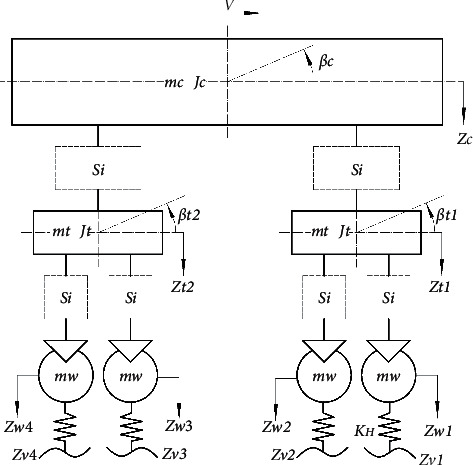
Side-view model of the high-speed train.

Vertical dynamic performance is studied in this paper, and a vertical model of high-speed train is taken as the research object, which consists of one vehicle body, two bogies, and four wheelsets, as shown in Figure 1. Suspensions between the wheelsets and bogies are primary suspensions and the ones between the body and bogies are secondary suspensions. The vertical motion of the high-speed train is weakly coupled with the lateral motion, which can be ignored when vertical vibration is mainly focused [16]. So, a 10-degree-of-freedom (DOF) train is modelled, as shown in Table 1, including the transitional motions and pitch motions of train body and both bogies, transitional motions of 4 wheelsets.

More details can be found in Figure 1. *V* is the running speed of the high-speed train; *m*_*c*_, *m*_*t*_, and *m*_*w*_ denote the mass of train body, bogies, and wheelsets, respectively. *J*_*c*_ and *J*_*t*_ are the pitch inertia of train body and bogies; *β*_*c*_, *β*_*t*1_, and *β*_*t*2_ denote the pitch angle of the train body, front and rear bogie; *z*_*c*_, *z*_*t*1_, and *z*_*t*2_ are the vertical displacements of train body, front and rear bogie, respectively. As for *z*_*w*1_, *z*_*w*2_, *z*_*w*3_, and *z*_*w*4_, they are the vertical displacements of four wheelsets. S*i* denotes the different suspension layouts, where *i* = 1, 2, 3, 4, 5, and in the analysis, the same suspension structure with inerter is applied to the primary suspension and secondary suspension of the high-speed train at the same time. *l*_*c*_ and *l*_*t*_ are the semilongitudinal spacing of secondary suspension and wheelsets.

*z*_*v*1_, *z*_*v*2_, *z*_*v*3_, and *z*_*v*4_ are the track inputs at different wheelsets, and the following relationship exists between them:

(1) $$\begin{array}{c} z_{v_{1}}\left(t\right)=z_{v_{2}}\left(t+\frac{2l_{t}}{V}\right)=z_{v_{3}}\left(t+\frac{2l_{c}}{V}\right)=z_{v_{4}}\left[t+\frac{2\left(l_{c}+l_{t}\right)}{V}\right], \end{array}$$

where *V* is running speed.

Considering that the track inputsare transmitted to the wheelsets through wheel-rail contacts, the wheel-rail contact stiffness *K*_*H*_ is introduced in the vertical plane. The wheel-rail vertical force is determined by the Hertzian nonlinear elastic contact theory, which has been applied for the wheel-rail contact for years due to its easy calculation and high accuracy. By calculating the force between the wheel and track, the wheel-rail contact stiffness can be approximately linearized as follows [17, 18]:

(2) $$\begin{array}{c} K_{H}=\frac{1.5\sqrt[{3}]{P_{0}}}{G}, \end{array}$$

where *P*_0_ is the wheel-rail static force and *G* is the wheel-rail contact constant, and for the conical tread wheel in this paper,

(3) $$\begin{array}{c} G=4.57R^{-0.149}\times 10^{-8}\;{\text{m}/\text{N}}^{2/3}. \end{array}$$

And in this paper, model CRH380A high-speed train is selected, and the parameters are shown in Table 2.

Equations of motions are established by Newton Euler Approach, as shown in formula (4). The subscripts *p* and *s* of *F* represent the primary and secondary suspensions, respectively, so, *F*_*p*1_, *F*_*p*2_, *F*_*p*3_, and *F*_*p*4_ denote the vertical forces of the four primary suspensions corresponding to the four wheelsets. *F*_*s*1_ and *F*_*s*2_ represent the vertical force of two secondary suspensions corresponding to two bogies.

(4) $$\begin{array}{c} \left\{\begin{array}{c} m_{c}{\overset{¨}{z}}_{c}=F_{s_{1}}+F_{s_{2}},\\ J_{c}{\overset{¨}{\beta }}_{c}=l_{c}\left(F_{s_{1}}-F_{s_{2}}\right),\\ m_{t}{\ddot{z}}_{t_{1}}=F_{p_{1}}+F_{p2}-F_{s_{1}},\\ J_{t}{\overset{¨}{\beta }}_{t_{1}}=l_{t}\left(F_{p_{1}}-F_{p_{2}}\right),\\ m_{t}{\overset{¨}{z}}_{t_{2}}=F_{p_{3}}+F_{p_{4}}-F_{s_{2}},\\ J_{t}{\ddot{\beta }}_{t_{2}}=l_{t}\left(F_{p_{3}}-F_{p_{4}}\right),\\ m_{w}{\ddot{z}}_{w_{1}}=F_{p_{1}}-K_{H}z_{v_{1}},\\ m_{w}{\ddot{z}}_{w_{2}}=F_{p_{1}}-K_{H}z_{v_{2}},\\ m_{w}{\ddot{z}}_{w_{3}}=F_{p_{3}}-K_{H}z_{v_{3}},\\ m_{w}{\ddot{z}}_{w_{4}}=F_{p_{4}}-K_{H}z_{v_{4}}. \end{array}\right. \end{array}$$

Considering formula (4) and the vertical forces expressions of the suspensions in Appendix, the system dynamics differential equation can be derived as

(5) $$\begin{array}{c} M_{0}\ddot{Z}+C_{0}\dot{Z}+K_{0}Z=K_{f}Q. \end{array}$$

*M *_0_, *C*_0_, *K*_0_, and *K*_*f*_ are the inertia matrix, the damping matrix, the stiffness matrix, and the input coefficient matrix, respectively. Also $\ddot{Z}$, $\dot{Z}$, and *Z* are the acceleration, velocity, and displacement vectors, respectively, while the input vector is expressed as *Q* = [*z*_*v*1_; *z*_*v*2_; *z*_*v*3_; *z*_*v*4_]^*T*^. After defining the output variable *y* of the system, the state space expression can be expressed as

(6) $$\begin{array}{c} \left\{\begin{array}{c} \dot{x}=Ax+Bu,\\ y=Cx+Du\;, \end{array}\right. \end{array}$$

where $x={\left[\begin{array}{cc} Z & \dot{Z} \end{array}\;\right]}^{T}$, and *Z*=[*z*_*c*_, *β*_*c*_, *z*_*t*_1__, *β*_*t*_1__, *z*_*t*_2__, *β*_*t*_2__, *z*_*w*_1__, *z*_*w*_2__, *z*_*w*_3__, *z*_*w*_4__]^*T*^.

The vector *u* is used to define the input from the track, which is track irregularity.

### 2.2. ISD Suspension Layouts

**Figure 2:**
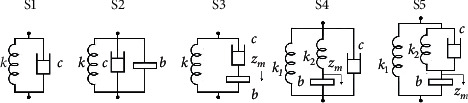
Suspension layouts.

Five different layouts are introduced in this section. As shown in Figure 2, S1 is the traditional suspension layout, which is a spring and a damper connected in parallel. It is the basic layout as a comparison with others. S2 and S3 have one spring, one damper, and one inerter. S2 has all 3 elements connected in parallel while S3 has the damper and the inerter series connected first, and then the spring parallelly connects with them. What is more, S4 and S5 add an extra supporting spring for safety reason. Besides the supporting spring, in S4, another spring and an inerter are connected in series first, and then the damper connects with them in parallel. In S5, a set of parallel connected springs and dampers connect with the inerter in series, with the supporting spring connecting in parallel with them all. All the ISD layouts are of low complexity to meet the actual requirements, such as the installation space or easy implementation.

Since five layouts are introduced, all the vertical forces of the suspensions can be expressed by equations, as in Appendix. As mentioned above, the subscripts *p* and *s* are used to distinguish the primary and secondary suspensions when the equation of the suspensions are established. For example, the spring stiffness coefficients of primary and secondary suspension are represented as *k*_*p*_ and *k*_*s*_, respectively.

What needs to be mentioned, in S3, S4, and S5 of Figure 2, an extra degree of freedom *z*_*m*_, the vertical displacements between elements of the series connection, is introduced to complete the system degree of freedom. In the equations, *z*_*mpi*_ (*i* = 1, 2, 3, 4) and *z*_*msj*_ (*j* = 1, 2) indicate the primary or secondary suspension, and the number represents the corresponding bogies or wheelsets. In this case, when the suspensions with S3, S4, or S5 are explored, the DOF of the whole vertical model will increase to 16 with 6 vertical displacements between elements of the series connection in consideration and the equations of the force equilibrium at *z*_*m*_ have to be supplemented, as shown in equations of S3, S4, and S5 in Appendix.

## 3. Optimization of Suspension Parameters

To make the riding comfort better for the modern track transportation, the parameters of the aforementioned suspension layouts are optimized. The intelligent optimization method of particle swarm optimization (PSO) algorithm is introduced to complete the optimization in this section.

### 3.1. Optimization Objectives

In this paper, the influence of suspension layouts with inerters on riding comfort is mainly focused. The vertical acceleration, pitch acceleration, and vertical displacement of the train body are selected as the optimization objectives. The smaller the value of objective function is, the better the performance of the riding comfort will be. All the indexes are calculated as follows.

The root-mean-square value (RMS) of train body vertical acceleration *J*_1_:

(7) $$\begin{array}{c} J_{1}=\sqrt{\frac{1}{T}\int_{0}^{T}{\ddot{z}}_{c}^{2}\text{ d}t}. \end{array}$$

The RMS of train body pitch acceleration *J*_2:_

(8) $$\begin{array}{c} J_{2}=\sqrt{\frac{1}{T}\int_{0}^{T}{\ddot{\beta }}_{c}^{2}\text{ d}t}. \end{array}$$

The RMS of train body vertical displacement *J*_3:_

(9) $$\begin{array}{c} J_{3}=\sqrt{\frac{1}{T}\int_{0}^{T}z_{c}^{2}\text{ d}t}, \end{array}$$

where *T* is time of the running period.

The objective function of optimization can be written as

(10) $$\begin{array}{c} Q=\frac{J_{1}}{J_{1}^{*}}+\frac{J_{2}}{J_{2}^{*}}+\frac{J_{3}}{J_{3}^{*}}, \end{array}$$

where *J*_*i*_^*∗*^ is the RMS of the corresponding values of the traditional suspension S1 under the same excitation.

### 3.2. Optimization Method

**Figure 3:**
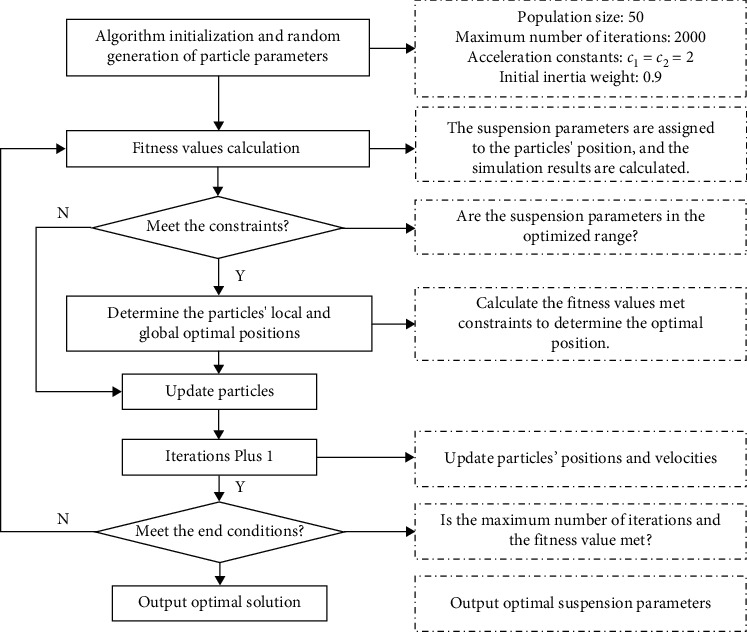
The procedure of PSO.

PSO is an intelligent algorithm, which originated from the simulation of bird swarm foraging behaviour, and its optimization ability is strong and easy to realize [19] and it has proven efficiency in the suspension optimization [14, 20]. To find the optimal parameters of the high-speed train suspensions for different layouts, the basic setting and the procedure of PSO in this paper are shown as in Figure 3.

As mentioned in Section 1, random track irregularity excitation is taken as the system input for the analysis of the performance; normally it is a statistical characteristic obtained by field measurement. In this paper, the German high-speed track spectrum is selected as the input. Since the normal running speed of the high-speed train is above 300 km/h, the track irregularity PSDs of Germany with low disturbance is [21]

(11) $$\begin{array}{c} S_{V}\left(\Omega \right)=\frac{A_{V}\Omega_{c}^{2}}{\left(\Omega^{2}+\Omega_{r}^{2}\right)\left(\Omega^{2}+\Omega_{c}^{2}\right)}. \end{array}$$

**Figure 4:**
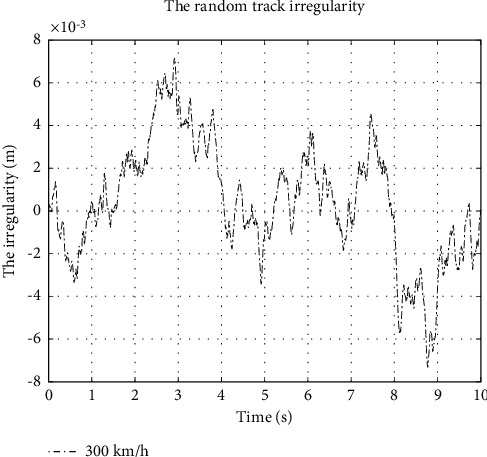
The track irregularity at the speed of 300 km/h.

Then the time domain expression is

(12) $$\begin{array}{c} {\ddot{z}}_{0}=-v\left(\Omega_{c}+\Omega_{r}\right){\dot{z}}_{0}-v^{2}\Omega_{c}\Omega_{r}z_{0}+\sqrt{2\pi A_{V}\Omega_{c}^{2}v^{3}}\omega \left(t\right), \end{array}$$

where *z*_0_ is the displacement of random track irregularity excitation and ${\dot{z}}_{0}$, ${\ddot{z}}_{0}$ are the first and second derivatives of *z*_0_, respectively. According to [21], Ω is the spatial frequency of track irregularity. *A*_*V*_ is the roughness constant, which is 4.032 × 10^−7^ mrad for low disturbance. The cut-off frequency Ω_*c*_ is 0.8246 rad/m while Ω_*r*_ is 0.0206 rad/m;*v* is the velocity of the high-speed train in m/s; *ω* (*t*) is a source of white noise disturbance and *t* is time variable. The time-domain irregularity can be calculated with these parameters, as shown in Figure 4. It is the irregularity when the running speed is 300 km/h.

In order to make the suspension system parameters optimization results reasonable and convenient for actual adjustment, the optimization ranges for the spring stiffness and damping float are 30% up and down of the original train suspension parameters, and the inertance optimization range is 1 to 50000 kg. The ranges of the optimal value are obtained as expressed in Table 3.

### 3.3. Optimization Results

The optimization is carried out when the train travels at the speed of 350 km/h, which is the normal running speed in practice. The optimal results are calculated in MATLAB as shown in Table 4. S1 in Table 4 takes the parameters of original set for comparison.

All the parameters will be taken to do the analysis in the next section to find out which layout shows the best performances in the vertical performance.

## 4. Performance Analysis

In this section, the vertical performances of the high-speed train at different speeds with all types of suspension layouts are analysed. The transitional and pitch acceleration of the train body, as well as the displacement of the train body, are discussed.

### 4.1. Response Analysis in Time Domain

The optimized suspension parameters are substituted into the high-speed train model and the model is simulated in MATLAB with the State Space Method as well.

**Figure 5:**
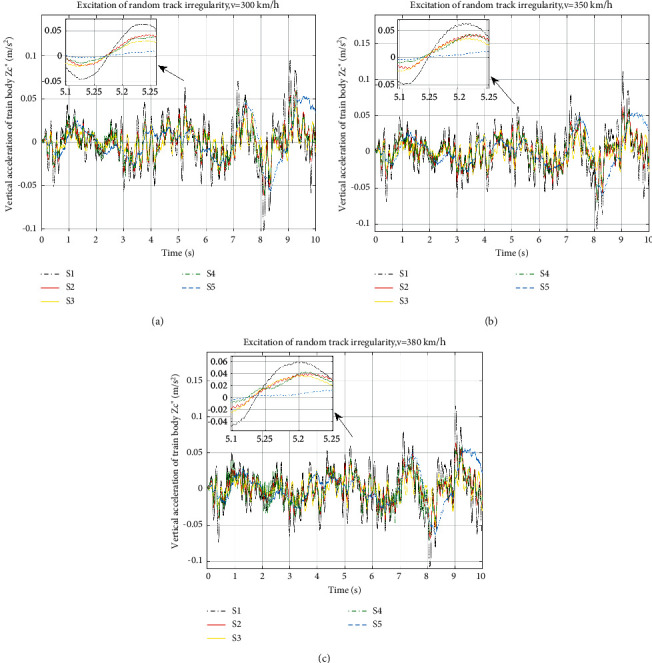
Vertical acceleration of train body at different speeds: (a) vertical acceleration of train body at 300 km/h, (b) vertical acceleration of train body at 350 km/h, and (c) vertical acceleration of train body at 380 km/h.

In the simulation, different speeds of 300 km/h, 350 km/h, and 380 km/h are selected.  Figure 5 shows the vertical acceleration of the train body in time domain. All the suspension layouts are analysed at 3 speeds; Figure 5(a) shows the response at 300 km/h, Figure 5(b) 350 km/h, and Figure 5(c) 380 km/h. The RMS values of train body vertical acceleration and their improvements are shown in Table 5.

From Figure 5, the vertical accelerations of different suspension layouts have the same trend of the curves. However, in all the figures, it can be found that, comparing with the black dot dash curve of the traditional layout S1, the amplitude of the vibration of all the ISD layouts is lowered. It indicates that no matter how the layouts connected, inerters do benefit the suspension in vertical vibration performances. It can be proven in more detail from Table 5 that, compared with the traditional suspension S1 at 350 km/h, which is the optimization running condition, the RMS of the train body vertical acceleration with S2–S5 decreases by 34.69%, 52.54%, 36.53%, and 22.19%, respectively. The improvement is huge especially for S3, which promotes about half of the vibration attenuation performance.

On the other hand, when the speed is 300 km/h, the RMS values of train body vertical acceleration with 4 ISD layouts are optimized by 32.70%, 53.09%, 33.61%, and 19.40%, respectively. The corresponding values are 35.24%, 52.00%, 33.60%, and 22.74%, respectively, when the speed is 380 km/h. We can see that benefits of performance still hold even at different speeds, and for the same type of layout, improvements at different speeds are basically the same. All the layouts with inerters are effective to the vertical acceleration of the train body, of which S3 has the best reduction of the vertical body acceleration. We can also tell that with the increase of the speed, that the amplitude of the acceleration increases as well.

**Figure 6:**
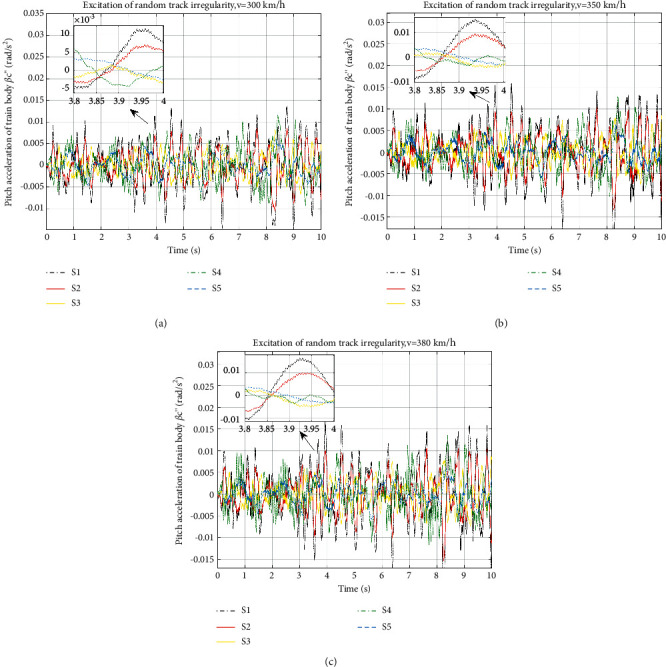
Pitch acceleration of train body under different speeds: (a) pitch acceleration of train body at 300 km/h, (b) pitch acceleration of train body at 350 km/h, and (c) pitch acceleration of train body at 380 km/h.

The pitch accelerations of the train body at speeds of 300 km/h, 350 km/h, and 380 km/h in time domain are shown in Figure 6 and the RMS of the acceleration and improvements are shown in Table 6.

Figure 6 reflects the pitch accelerations of different layouts at different speeds. Curves are of the same trends and amplitudes level. Curves of ISD layouts sometimes show worse vibration than S1, such as S4 at 7.5 s in all speeds. By analysing the RMS values in Table 6, it can be concluded that the pitch accelerations also show a similar improvement effect to the vertical body accelerations. All ISD layouts with inerters benefit the train body pitch acceleration, and for the same type of layout, improvements at different speeds are basically the same as well. In the performance of pitch acceleration, S5 is the best; at 380 km/h, an amazing 68.27% improvement is given. S3 shows the second best improvement, which is about 50% of promotion, as shown in Table 6.

**Figure 7:**
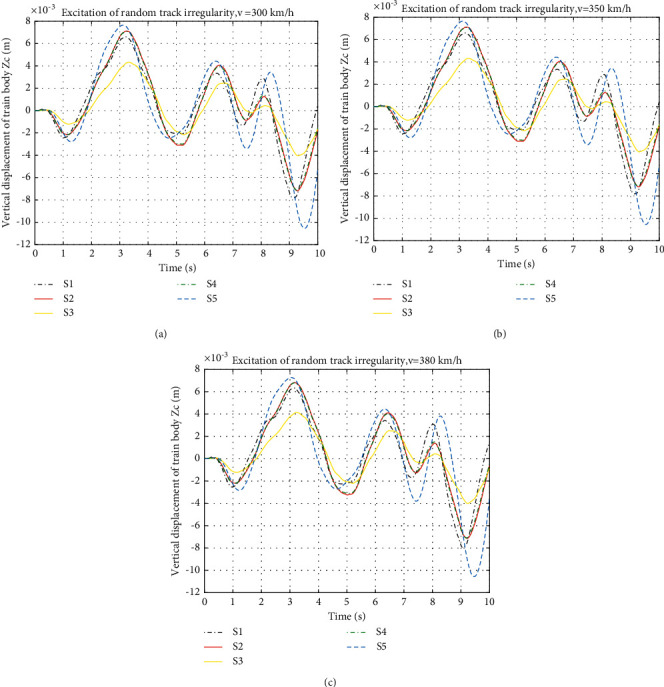
Vertical displacement of train body under different speeds: (a) vertical displacement of train body at 300 km/h, (b) vertical displacement of train body at 350 km/h, and (c) vertical displacement of train body at 380 km/h.

The vertical displacements of the train body at speeds of 300 km/h, 350 km/h, and 380 km/h in time domain are shown in Figure 7 and the RMS of the displacement and improvements are shown in Table 7.

Figure 7 shows the train body vertical displacement of different layouts at different speeds. Compared with S1, S3 can effectively reduce the amplitude of the vertical displacement while the other three layouts show negative effects. The displacement at some peaks is much larger than S1. By analysing the RMS values in Table 7, it can be seen that S2 and S4 show deterioration in performance of vertical displacement of 3.58% and 2.38%, respectively, while S5 shows the worst deterioration of 28.5%. Only S3 suspension has an improvement of 38% compared with S1.

For both the vertical and pitch acceleration performances, it shows that benefits all existed for all layouts at different speeds, and even the optimization is carried out only at the speed of 350 km/h. The improvement is basically the same when the same type of layout is used at different speeds. In the time domain, the ISD layouts with inerters do benefit the riding comfort. And with these three indexes in consideration, S3 shows the best improvement in vertical acceleration and vertical displacement while S5 shows the best improvement in pitch acceleration. For the displacement of the train body, only S3 shows an improvement compared with S1; the others deteriorate the amplitudes in different levels.

### 4.2. Analysis in Frequency Domain

**Figure 8:**
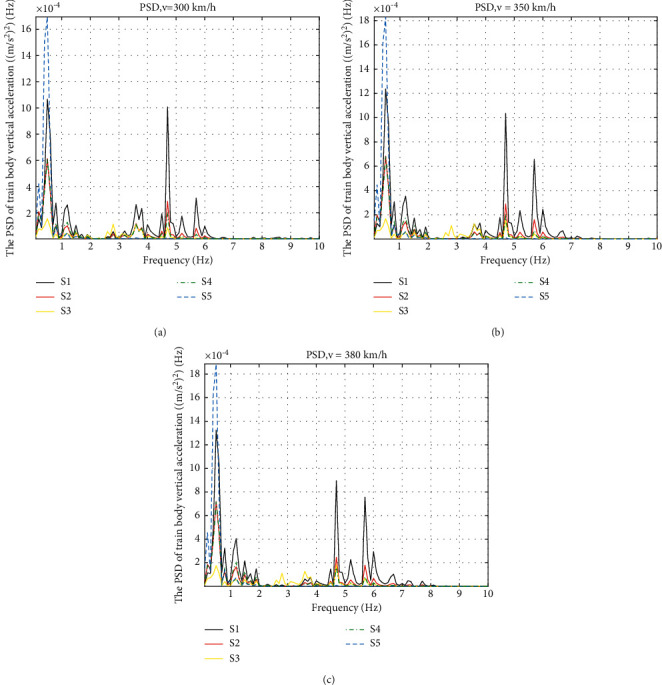
PSD of train body vertical acceleration at different speeds: (a) PSD of train body vertical acceleration at 300 km/h, (b) PSD of train body vertical acceleration at 350 km/h, and (c) PSD of train body vertical acceleration at 380 km/h.

**Figure 9:**
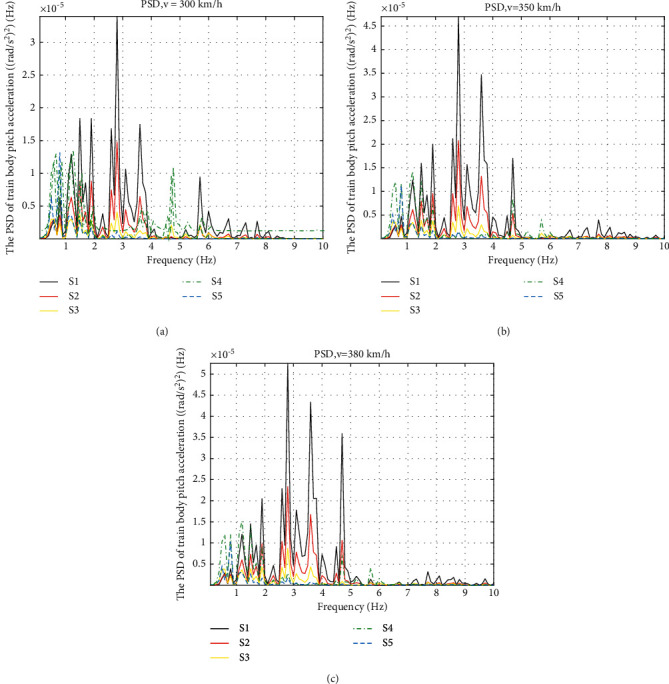
PSD of train body pitch acceleration at different speeds: (a) PSD of train body pitch acceleration at 300 km/h, (b) PSD of train body pitch acceleration at 350 km/h, and (c) PSD of train body pitch acceleration at 380 km/h.

Compared to the displacement, the performance of the accelerations needs to be studied more. Analysis of accelerations in frequency domain is discussed next. The PSD of the train body vertical and pitch acceleration are shown in Figures 8 and 9.

By comparing the PSD of the train body vertical acceleration with different suspension layouts in Figure 8, it can be seen that the PSD curves present basically the same trend at three speeds. Taking the speed of 350 km/h in Figure 8(b) as an example, the natural frequency of the five curves is the same at 0.4 Hz; S2, S3, and S4 lower the amplitude at the natural frequency, but S5 gives a deterioration of about 46%. In the range of 0.8–2.5 Hz and 4–10 Hz, all the ISD layout curves show a lower amplitude, especially S3 has a biggest drop of the amplitude. However, around 3-4 Hz, S3 introduces some noise in that area.

The PSD curves of the train body pitch acceleration are shown in Figure 9. Similar conclusions can be drawn at the different speeds. Again, we analyse the condition of 350 km/h in Figure 9(b). The natural frequency of S1, S2, and S3 is 2.8 Hz, but S4 and S5 lower their natural frequencies to 1.2 Hz and 0.7 Hz. The amplitude of the PSD in the range of 1–4 Hz is lowered by the ISD layouts. And S4 shows the best effect on lowering the amplitude of the PSD in this range, which is the most sensitive frequency range to the humans of riding comfort. However, for the larger rang of 1–10 Hz, S3 shows the best average attenuation. It also gives the lowest amplitude at the natural frequency.

The comprehensive analysis shows that installing suspension with ISD layouts in the primary and secondary suspensions of the high-speed trains at the same time have better performance for riding comfort in both time and frequency domain, and among S1 to S5, S3 is assessed to be the best choice in every aspect, being also of low-complexity and easy to realize.

## 5. Simulation and Analysis with Nonlinear Inerter

The performance benefits are analysed in Section 3. The analysis results obtained by the MATLAB simulation system provide a reference for choosing suspension layouts. In this section, the nonlinearity of inerters in ISD layout will be discussed using RecurDyn simulation approach. The vertical and pitch accelerations of the train body are studied with the friction of inerters in consideration.

### 5.1. Virtual Prototyping Modelling

Nonlinearity is a factor that cannot be eliminated in reality, so with the help of simulating software RecurDyn, a simulation is studied with a nonlinear inerter.

**Figure 10:**
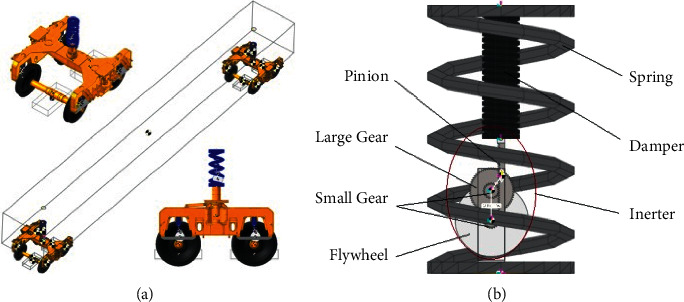
Virtual prototype model of the high-speed train with S3. (a) The whole model. (b) Modeling of S3 layout.

Firstly, a virtual prototype model of the high-speed train is modelled in RecurDyn, which contains one train body, two bogies, and four wheelsets as well in Figure 10(a). The traditional suspension S1 is established firstly with the purpose of model verification.

And according to the analysis above, the layout S3 has the best performance among the four proposed ISD layouts. A virtual prototype model is established based on S3 layout for the model suspension, where a rack-pinion inerter is introduced to construct S3 layout, as shown in Figure 10(b). The parameters are the same in Table 2 for the train and Table 4 for S3.

### 5.2. Model Verification

**Figure 11:**
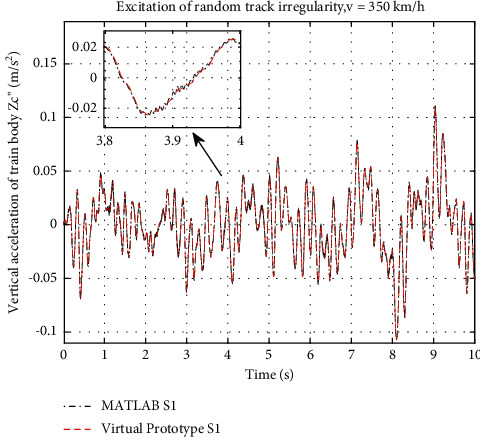
Vertical acceleration of train body.

Verification of the virtual prototype must be done before the simulation. Model with layout S1 is simulated first with the same track irregularity at 350 km/h as the input of the virtual system. The simulation results are shown in Figure 11 with the comparison of the mathematical result calculated in MATLAB.

It can be found that the vertical acceleration curve of the train body simulated by the virtual prototype shows a good agreement with the curve calculated by MATLAB, so it can be concluded that the virtual prototype model is believable. And the following nonlinearity study will be carried out based on this model.

### 5.3. Simulation and Analysis with Nonlinear Inerters

The influence of friction is one of the main nonlinearities of an inerter, in this section the rack-pinion inerter and its nonlinearity from friction are studied. The friction of the inerter is established in the virtual prototype model, which comes from the relative motion between the rotation of the gears and bearings, and the transition between the rack and frame. The friction coefficientsettings for the revolute joints and translation joints are shown in Table 8.

**Figure 12:**
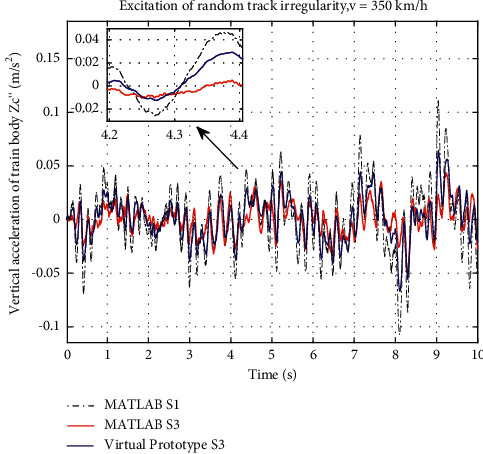
Vertical acceleration response curve of train body.

After the friction setting and the RecurDyn simulation, the curve of the vertical acceleration of train body employing S3 is drawn in blue curve in Figure 12. At the same time, curves from the traditional suspension and ideal S3 layout in Section 3 are taken as references, which are black-dot dash line and red line. From the comparison, the nonlinear curve is not as good as the ideal S3, but it still has benefit in the performance. The RMS of train body vertical acceleration of ideal S3 layout is optimized by 52.54% compared to the S1 layout, while about 33.31% is obtained for the nonlinear one.

**Figure 13:**
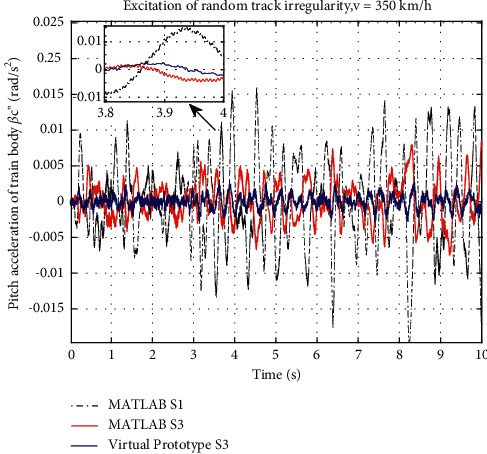
Pitch acceleration response curve of train body.

The pitch acceleration curve obtained in RecurDyn is shown in Figure 13. The amplitude of pitch acceleration curve of the nonlinear S3 is lower than that of ideal S3 simulating in Section 3 and the traditional layout. When S3 suspension considers the friction of inerters, it gets even better performance than the ideal S3 suspension in the pitch acceleration. From the RMS, the ideal S3 layout in the performance of pitch acceleration is optimized by 56.96% compared to S1. However, the nonlinear S3 suspension performs even better, which is improved by 85.04%.

**Figure 14:**
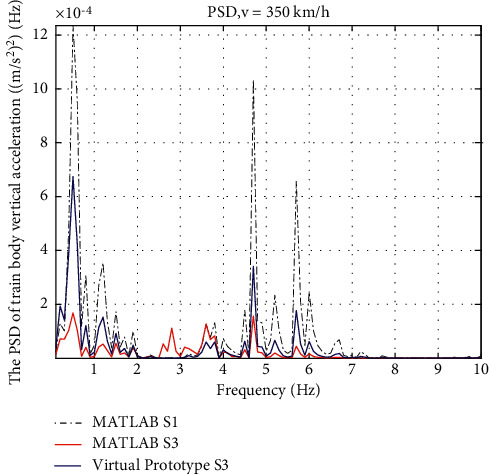
PSD of train body vertical acceleration.

**Figure 15:**
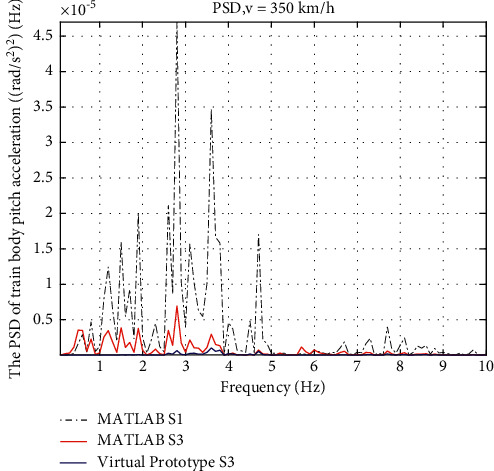
PSD of train body pitch acceleration.

Results are also shown in frequency domain. In Figure 14, the PSD of the vertical acceleration with friction shows a deterioration comparing to the ideal one, but the amplitude is still much lower than S1. In Figure 15, the benefits of friction give the lowest PSD curve of the pitch acceleration, which indicates that the friction resists the pitch motion of the train body in a good way.

The friction brings the nonlinearity to the inerter and the ISD suspension. The effect has two aspects: for the vertical acceleration, it deteriorates the performance of vibration attenuation but still holds the benefit compared with the traditional suspension. And, for the pitch acceleration of the train body, the introduction of friction improves more than the ideal ISD suspension. In reality, nonlinearity like friction cannot be ignored. The introduction of nonlinear factors in this virtual prototype makes the simulation more practical and can be a guidance for the engineering application in the design of the high-speed train ISD suspension.

## 6. Conclusions

With the assistance of the intelligent algorithm, this paper discuss the structure design of the train suspension. The riding comfort of a high-speed train is studied and suspension systems with the introduction of different ISD layouts are analysed.

1. A 10-DOF vertical model of high-speed train is established and 4 ISD layouts of suspension are introduced in this paper. The performances of riding comfort are studied in both time and frequency domain at different speeds.
2. With the intelligent method of PSO, the parameters of four new ISD suspension layouts are optimized. Compared with the traditional suspension, the four new types of suspension can improve the riding comfort of the train in both vertical and pitch accelerations, and S3 has the best performance in the comprehensive assessment. What is more, the ISD layouts hold the performance benefits at different running speeds of the train.
3. A virtual prototype simulation is carried out in RecurDyn software. The friction of inerters is considered in the S3 layout. The results show that with the friction, the ISD suspension can still improve the riding comfort of the high-speed train.

All the results concluded in this paper can be a useful guidance for the future train suspension design, which will give a better riding comfort for the passengers and serve the intelligent rail transportation system update well.

## Figures and Tables

**Table 1:** DOF of the train.

Component	Transitional motion	Pitch motion
Train body	*z *_*c*_	*β *_*c*_
Bogies	*z *_*ti*_ (*i* = 1∼2)	*β *_*ti*_ (*i* = 1∼2)
Wheelsets	*z *_*wi*_ (*i* = 1∼4)	—

**Table 2:** Parameters of the 10-DOF high-speed train vertical model.

Symbol	Parameter	Unit	Value
*V*	Speed	m/s	97.22
*m *_*c*_	Train body mass	kg	43862.5
*J *_*c*_	Train body pitch inertia	kg·m^2^	1654000
*m *_*t*_	Bogie mass	kg	2400
*J *_*t*_	Bogie pitch inertia	kg·m^2^	1314
*m*_*w*_	Wheelset mass	kg	1850
*R*	Wheel radius	m	0.43
*l *_*c*_	Semilongitudinal spacing of secondary suspension	m	8.75
*l*t	Semilongitudinal spacing of wheelsets	m	1.25

**Table 3:** The range of the optimal parameters value.

Optimization parameters	Original parameters	Lower limit	Upper limit
*k *_*p*_/N/m	1176000	823200	1528800
*k *_*s*_/N/m	190000	133000	247000
*c *_*p*_/Ns/m	13000	9100	16900
*c *_*s*_/Ns/m	40000	28000	52000
*b *_*p*_/kg	—	1	50000
*b *_*s*_/kg	—	1	50000

**Table 4:** Parameters optimization results.

Layouts	Optimization results (N/m, Ns/m, kg)
S1	*k *_*p*_ = 1176000, *k*_*s*_ = 190000 *c*_*p*_ = 13000, *c*_*s*_ = 40000
S2	*k *_*p*_ = 823200, *k*_*s*_ = 133000, *c*_*p*_ = 16900 *c*_*s*_ = 28000, *b*_*p*_ = 17, *b*_*s*_ = 13
S3	*k *_*p*_ = 823200, *k*_*s*_ = 133000, *c*_*p*_ = 16899 *c*_*s*_ = 28000, *b*_*p*_ = 988, *b*_*s*_ = 38250
S4	*k *_*p*1_ = 823200, *k*_*p*2_ = 1528800, *k*_*s*1_ = 133000 *k*_*s*2_ = 246999, *c*_*p*_ = 16899, *c*_*s*_ = 28000 *b*_*p*_ = 235, *b*_*s*_ = 17
S5	*k *_*p*1_ = 823200, *k*_*p2*_ = 1528799, *k*_*s*1_ = 173117 *k*_*s*2_ = 133000, *c*_*p*_ = 9100, *c*_*s*_ = 28000 *b*_*p*_ = 34239, *b*_*s*_ = 108

**Table 5:** The RMS of train body vertical acceleration (m/s^2^) and improvement (%).

Layouts		Speeds		
		300 km/h	350 km/h	380 km/h
S1	*J*_1_	0.0277	0.0299	0.0307
S2	*J*_1_	0.0186	0.0195	0.0199
	Impro.	32.70%	34.69%	35.24%
S3	*J*_1_	0.0130	0.0142	0.0147
	Impro.	53.09%	52.54%	52.00%
S4	*J*_1_	0.0184	0.0190	0.0204
	Impro.	33.61%	36.53%	33.60%
S5	*J*_1_	0.0223	0.0233	0.0237
	Impro.	19.40%	22.19%	22.74%

**Table 6:** The RMS of train body pitch acceleration (rad/s^2^) and improvement (%).

Layouts		Speeds		
		300 km/h	350 km/h	380 km/h
S1	*J*_2_	0.0054	0.0062	0.0067
S2	*J*_2_	0.0035	0.0040	0.0042
	Impro.	34.56%	34.83%	35.69%
S3	*J*_2_	0.0024	0.0027	0.0028
	Impro.	55.37%	56.96%	57.76%
S4	*J*_2_	0.0037	0.0038	0.0042
	Impro.	31.77%	37.79%	37.41%
S5	*J*_2_	0.0022	0.0022	0.0021
	Impro.	58.19%	64.74%	68.27%

**Table 7:** The RMS of train body vertical displacement (m) and improvement (%).

Layouts		Speeds		
		300 km/h	350 km/h	380 km/h
S1	*J*_3_	0.003288	0.003254	0.003229
S2	*J*_3_	0.003440	0.003384	0.003345
	Impro.	−4.64%	−4.00%	−3.58%
S3	*J*_3_	0.002059	0.002021	0.001993
	Impro.	37.38%	37.92%	38.27%
S4	*J*_3_	0.003399	0.003345	0.003306
	Impro.	−3.38%	−2.77%	−2.38%
S5	*J*_3_	0.004171	0.004168	0.004149
	Impro.	−26.88%	−28.05%	−28.50%

**Table 8:** Friction coefficient settings of virtual prototype model.

Type	Rack friction	Bearing friction
Static friction coefficient	0.12	0.0015
Dynamic friction coefficient	0.10	0.0010

## Data Availability

The data in this study are calculated by the author. The data used to support the findings of this study are available from the corresponding author upon request.

## Appendix

### Vertical Forces of 5 Layouts

(A.1)
$$\begin{array}{c} S1\left\{\begin{array}{c} F_{s_{1}}=k_{s}\left(z_{c}+l_{c}\beta_{c}-z_{t_{1}}\right)+c_{s}\left({\dot{z}}_{c}+l_{c}{\dot{\beta }}_{c}-{\dot{z}}_{t_{1}}\right),\\ F_{s_{2}}=k_{s}\left(z_{c}-l_{c}\beta_{c}-z_{t_{1}}\right)+c_{s}\left({\dot{z}}_{c}-l_{c}{\dot{\beta }}_{c}-{\dot{z}}_{t_{1}}\right),\\ F_{p_{1}}=k_{p}\left(z_{t_{1}}+l_{t}\beta_{t_{1}}-z_{w_{1}}\right)+c_{p}\left({\dot{z}}_{t_{1}}+l_{t}{\dot{\beta }}_{t_{1}}-{\dot{z}}_{w_{1}}\right),\\ F_{p_{2}}=k_{p}\left(z_{t_{1}}-l_{t}\beta_{t_{1}}-z_{w_{2}}\right)+c_{p}\left({\dot{z}}_{t_{1}}-l_{t}{\dot{\beta }}_{t_{1}}-{\dot{z}}_{w_{2}}\right),\\ F_{p_{3}}=k_{p}\left(z_{t_{2}}+l_{t}\beta_{t_{2}}-z_{w_{3}}\right)+c_{p}\left({\dot{z}}_{t_{2}}+l_{t}{\dot{\beta }}_{t_{2}}-{\dot{z}}_{w_{3}}\right),\\ F_{p_{4}}=k_{p}\left(z_{t_{2}}-l_{t}\beta_{t_{2}}-z_{w_{4}}\right)+c_{p}\left({\dot{z}}_{t_{2}}-l_{t}{\dot{\beta }}_{t_{2}}-{\dot{z}}_{w_{4}}\right), \end{array}\right.\\ S2\left\{\begin{array}{c} F_{s1}=k_{s}\left(z_{c}+l_{c}\beta_{c}-z_{t1}\right)+c_{s}\left({\dot{z}}_{c}+l_{c}{\dot{\beta }}_{c}-{\dot{z}}_{t1}\right)+b_{s}\left({\ddot{z}}_{c}+l_{c}{\ddot{\beta }}_{c}-{\ddot{z}}_{t1}\right),\\ F_{s2}=k_{s}\left(z_{c}-l_{c}\beta_{c}-z_{t2}\right)+c_{s}\left({\dot{z}}_{c}-l_{c}{\dot{\beta }}_{c}-{\dot{z}}_{t2}\right)+b_{s}\left({\ddot{z}}_{c}-l_{c}{\ddot{\beta }}_{c}-{\ddot{z}}_{t2}\right),\\ F_{p1}=k_{p}\left(z_{t1}+l_{t}\beta_{t1}-z_{w1}\right)+c_{p}\left({\dot{z}}_{t1}+l_{t}{\dot{\beta }}_{t1}-{\dot{z}}_{w1}\right)+b_{p}\left({\ddot{z}}_{t1}+l_{t}{\ddot{\beta }}_{t1}-{\ddot{z}}_{w1}\right),\\ F_{p2}=k_{p}\left(z_{t1}-l_{t}\beta_{t1}-z_{w2}\right)+c_{p}\left({\dot{z}}_{t1}-l_{t}{\dot{\beta }}_{t1}-{\dot{z}}_{w2}\right)+b_{p}\left({\ddot{z}}_{t1}-l_{t}{\ddot{\beta }}_{t1}-{\ddot{z}}_{w2}\right),\\ F_{p3}=k_{p}\left(z_{t2}+l_{t}\beta_{t2}-z_{w3}\right)+c_{p}\left({\dot{z}}_{t2}+l_{t}{\dot{\beta }}_{t2}-{\dot{z}}_{w3}\right)+b_{p}\left({\ddot{z}}_{t2}+l_{t}{\ddot{\beta }}_{t2}-{\ddot{z}}_{w3}\right),\\ F_{p4}=k_{p}\left(z_{t2}-l_{t}\beta_{t2}-z_{w4}\right)+c_{p}\left({\dot{z}}_{t2}-l_{t}{\dot{\beta }}_{t2}-{\dot{z}}_{w4}\right)+b_{p}\left({\ddot{z}}_{t2}-l_{t}{\ddot{\beta }}_{t2}-{\ddot{z}}_{w4}\right), \end{array}\right.\\ S3\left\{\begin{array}{c} F_{s1}=k_{s}\left(z_{c}+l_{c}\beta_{c}-z_{t1}\right)+c_{s}\left({\dot{z}}_{c}+l_{c}{\dot{\beta }}_{c}-{\dot{z}}_{ms1}\right)=k_{s}\left(z_{c}+l_{c}\beta_{c}-z_{t1}\right)+b_{s}\left({\ddot{z}}_{ms1}-{\ddot{z}}_{t1}\right),\\ F_{s2}=k_{s}\left(z_{c}-l_{c}\beta_{c}-z_{t2}\right)+c_{s}\left({\dot{z}}_{c}-l_{c}{\dot{\beta }}_{c}-{\dot{z}}_{ms2}\right)=k_{s}\left(z_{c}-l_{c}\beta_{c}-z_{t2}\right)+b_{s}\left({\ddot{z}}_{ms2}-{\ddot{z}}_{t2}\right),\\ F_{p1}=k_{p}\left(z_{t1}+l_{t}\beta_{t1}-z_{mp1}\right)+c_{p}\left({\dot{z}}_{t1}+l_{t}{\dot{\beta }}_{t1}-{\dot{z}}_{mp1}\right)=k_{p}\left(z_{t1}+l_{t}\beta_{t1}-z_{mp1}\right)+b_{p}\left({\ddot{z}}_{mp1}-{\ddot{z}}_{w1}\right),\\ F_{p2}=k_{p}\left(z_{t1}-l_{t}\beta_{t1}-z_{mp2}\right)+c_{p}\left({\dot{z}}_{t1}-l_{t}{\dot{\beta }}_{t1}-{\dot{z}}_{mp2}\right)=k_{p}\left(z_{t1}-l_{t}\beta_{t1}-z_{mp2}\right)+b_{p}\left({\ddot{z}}_{mp2}-{\ddot{z}}_{w2}\right),\\ F_{p3}=k_{p}\left(z_{t2}+l_{t}\beta_{t2}-z_{mp3}\right)+c_{p}\left({\dot{z}}_{t2}+l_{t}{\dot{\beta }}_{t2}-{\dot{z}}_{mp3}\right)=k_{p}\left(z_{t2}+l_{t}\beta_{t2}-z_{mp3}\right)+b_{p}\left({\ddot{z}}_{mp3}-{\ddot{z}}_{w3}\right),\\ F_{p4}=k_{p}\left(z_{t2}-l_{t}\beta_{t2}-z_{mp4}\right)+c_{p}\left({\dot{z}}_{t2}-l_{t}{\dot{\beta }}_{t2}-{\dot{z}}_{mp4}\right)=k_{p}\left(z_{t2}-l_{t}\beta_{t2}-z_{mp4}\right)+b_{p}\left({\ddot{z}}_{mp4}-{\ddot{z}}_{w4}\right), \end{array}\right.\\ S4\left\{\begin{array}{c} F_{s1}=k_{s1}\left(z_{c}+l_{c}\beta_{c}-z_{t1}\right)+c_{s}\left({\dot{z}}_{c}+l_{c}{\dot{\beta }}_{c}-{\dot{z}}_{t1}\right)+b_{s}\left({\ddot{z}}_{ms1}-{\ddot{z}}_{t1}\right)\\ =k_{s1}\left(z_{c}+l_{c}\beta_{c}-z_{t1}\right)+c_{s}\left({\dot{z}}_{c}+l_{c}{\dot{\beta }}_{c}-{\dot{z}}_{t1}\right)+k_{s2}\left(z_{c}+l_{c}\beta_{c}-z_{ms1}\right),\\ F_{s2}=k_{s1}\left(z_{c}-l_{c}\beta_{c}-z_{t2}\right)+c_{s}\left({\dot{z}}_{c}-l_{c}{\dot{\beta }}_{c}-{\dot{z}}_{t2}\right)+b_{s}\left({\ddot{z}}_{ms2}-{\ddot{z}}_{t2}\right)\\ =k_{s1}\left(z_{c}-l_{c}\beta_{c}-z_{t2}\right)+c_{s}\left({\dot{z}}_{c}-l_{c}{\dot{\beta }}_{c}-{\dot{z}}_{t2}\right)+k_{s2}\left(z_{c}-l_{c}\beta_{c}-z_{ms2}\right),\\ F_{p1}=k_{p1}\left(z_{t1}+l_{t}\beta_{t1}-z_{w1}\right)+c_{p}\left({\dot{z}}_{t1}+l_{t}{\dot{\beta }}_{t1}-{\dot{z}}_{w1}\right)+b_{p}\left({\ddot{z}}_{mp1}-{\ddot{z}}_{w1}\right)\\ =k_{p1}\left(z_{t1}+l_{t}\beta_{t1}-z_{w1}\right)+c_{p}\left({\dot{z}}_{t1}+l_{t}{\dot{\beta }}_{t1}-{\dot{z}}_{w1}\right)+k_{p2}\left(z_{t1}+l_{t}\beta_{t1}-z_{mp1}\right),\\ F_{p2}=k_{p1}\left(z_{t1}-l_{t}\beta_{t1}-z_{w2}\right)+c_{p}\left({\dot{z}}_{t1}-l_{t}{\dot{\beta }}_{t1}-{\dot{z}}_{w2}\right)+b_{p}\left({\ddot{z}}_{mp2}-{\ddot{z}}_{w2}\right)\\ =k_{p1}\left(z_{t1}-l_{t}\beta_{t1}-z_{w2}\right)+c_{p}\left({\dot{z}}_{t1}-l_{t}{\dot{\beta }}_{t1}-{\dot{z}}_{w2}\right)+k_{p2}\left(z_{t1}-l_{t}\beta_{t1}-z_{mp2}\right),\\ F_{p3}=k_{p1}\left(z_{t2}+l_{t}\beta_{t2}-z_{w3}\right)+c_{p}\left({\dot{z}}_{t2}+l_{t}{\dot{\beta }}_{t2}-{\dot{z}}_{w3}\right)+b_{p}\left({\ddot{z}}_{mp3}-{\ddot{z}}_{w3}\right)\\ =k_{p1}\left(z_{t2}+l_{t}\beta_{t2}-z_{w3}\right)+c_{p}\left({\dot{z}}_{t2}+l_{t}{\dot{\beta }}_{t2}-{\dot{z}}_{w3}\right)+k_{p2}\left(z_{t2}+l_{t}\beta_{t2}-z_{mp3}\right),\\ F_{p4}=k_{p1}\left(z_{t2}-l_{t}\beta_{t2}-z_{w4}\right)+c_{p}\left({\dot{z}}_{t2}-l_{t}{\dot{\beta }}_{t2}-{\dot{z}}_{w4}\right)+b_{p}\left({\ddot{z}}_{mp4}-{\ddot{z}}_{w4}\right)\\ =k_{p1}\left(z_{t2}-l_{t}\beta_{t2}-z_{w4}\right)+c_{p}\left({\dot{z}}_{t2}-l_{t}{\dot{\beta }}_{t2}-{\dot{z}}_{w4}\right)+k_{p2}\left(z_{t2}-l_{t}\beta_{t2}-z_{mp4}\right), \end{array}\right.\\ S5\left\{\begin{array}{c} F_{s1}=k_{s1}\left(z_{c}+l_{c}\beta_{c}-z_{t1}\right)+b_{s}\left({\ddot{z}}_{ms1}-{\ddot{z}}_{t1}\right)\\ =k_{s1}\left(z_{c}+l_{c}\beta_{c}-z_{t1}\right)+k_{s2}\left(z_{c}+l_{c}\beta_{c}-z_{mp1}\right)+c_{s}\left({\dot{z}}_{c}+l_{c}{\dot{\beta }}_{c}-{\dot{z}}_{mp1}\right),\\ F_{s2}=k_{s1}\left(z_{c}-l_{c}\beta_{c}-z_{t2}\right)+b_{s}\left({\ddot{z}}_{ms2}-{\ddot{z}}_{t2}\right)\\ =k_{s1}\left(z_{c}-l_{c}\beta_{c}-z_{t2}\right)+k_{s2}\left(z_{c}-l_{c}\beta_{c}-z_{mp2}\right)+c_{s}\left({\dot{z}}_{c}-l_{c}{\dot{\beta }}_{c}-{\dot{z}}_{mp2}\right),\\ F_{p1}=k_{p1}\left(z_{t1}+l_{t}\beta_{t1}-z_{w1}\right)+b_{p}\left({\ddot{z}}_{mp1}-{\ddot{z}}_{w1}\right)\\ =k_{p1}\left(z_{t1}+l_{t}\beta_{t1}-z_{w1}\right)+k_{p2}\left(z_{t1}+l_{t}\beta_{t1}-z_{mp1}\right)+c_{p}\left({\dot{z}}_{t1}+l_{t1}{\dot{\beta }}_{t1}-{\dot{z}}_{ms1}\right),\\ F_{p2}=k_{p1}\left(z_{t1}-l_{t}\beta_{t1}-z_{w2}\right)+b_{p}\left({\ddot{z}}_{mp2}-{\ddot{z}}_{w2}\right)\\ =k_{p1}\left(z_{t1}-l_{t}\beta_{t1}-z_{w2}\right)+k_{p2}\left(z_{t1}-l_{t}\beta_{t1}-z_{mp2}\right)+c_{p}\left({\dot{z}}_{t1}-l_{t1}{\dot{\beta }}_{t1}-{\dot{z}}_{mp2}\right),\\ F_{p3}=k_{p1}\left(z_{t2}+l_{t}\beta_{t2}-z_{w3}\right)+b_{p}\left({\ddot{z}}_{mp3}-{\ddot{z}}_{w3}\right)\\ =k_{p1}\left(z_{t2}+l_{t}\beta_{t2}-z_{w3}\right)+k_{p2}\left(z_{t2}+l_{t}\beta_{t2}-z_{mp3}\right)+c_{p}\left({\dot{z}}_{t2}+l_{t1}{\dot{\beta }}_{t2}-{\dot{z}}_{mp3}\right),\\ F_{p4}=k_{p1}\left(z_{t2}-l_{t}\beta_{t2}-z_{w4}\right)+b_{p}\left({\ddot{z}}_{mp4}-{\ddot{z}}_{w4}\right)\\ =k_{p1}\left(z_{t2}-l_{t}\beta_{t2}-z_{w4}\right)+k_{p2}\left(z_{t2}+l_{t}\beta_{t2}-z_{mp3}\right)+c_{p}\left({\dot{z}}_{t2}+l_{t1}{\dot{\beta }}_{t2}-{\dot{z}}_{mp3}\right). \end{array}\right. \end{array}$$
